# Diversification of Physical Activities: An Exploration of Provision Characteristics of Holistic Movement Practices in a Large Australian City

**DOI:** 10.3390/ijerph181910365

**Published:** 2021-10-01

**Authors:** Ineke Vergeer, Bojana Klepac-Pogrmilovic

**Affiliations:** 1 Centre for Health Research, University of Southern Queensland, Springfield Central, QLD 4300, Australia; 2 Mitchell Institute for Education and Health Policy, Victoria University, Melbourne, VIC 3011, Australia; Bojana.KlepacPogrmilovic@vu.edu.au

**Keywords:** mind–body, Yoga, Tai Chi, Qigong, conscious dance, physical activity diversity, mindful movement, sport management

## Abstract

Holistic movement practices (HMPs) are an emerging category of physical activity, contributing to the diversification of physical activity opportunities. Purposefully incorporating not only physical but also mental, social, and/or spiritual elements, HMPs have received limited research attention with respect to participation parameters. The purpose of this study was to begin to map HMPs’ participation potential by exploring the provision features of HMPs in Melbourne. Data were collected via internet searches, with a focus on events offered. Event features, including type, cost, duration, venue address, and target groups, were recorded. Associated neighbourhood characteristics were also explored by linking venue locations to selected census information. Provision was documented for Yoga and Pilates in central Melbourne (1011 events), for Tai Chi and Qigong (323 events), and for a range of smaller HMPs (149 events) across Greater Melbourne. Results indicated a wide range in provision features. Affinities with the holistic nature of HMPs were noticeable in venue choices and neighbourhood socio-demographics. Mention of specific target groups was infrequent. Results are discussed in light of implications for uptake. HMPs exemplify the increasing diversity of physical activity opportunities in modern-day societies. Further research to elucidate their place in the landscape of physical activities is warranted.

## 1. Introduction

While physical activity is often perceived as a leisure activity, it is also considered an important health behaviour [1,2], and as such, promotion of physical activity increasingly features as a public health priority [3]. In Australia, this priority is reflected in the broadening of the definition of sport in the most recent policy document “Sport 2030” [4], to include not only organised competitive sports but also a wide range of other physical activities, including informal, unstructured activities such as walking and swimming, as well as “new and evolving” forms of physical activity (p. 6). The focus in this paper is on some of these “new and evolving” forms of physical activity, in particular a variety of practices that can be referred to as “holistic movement practices” (HMPs) [5].

Sometimes categorised as “mind-body” disciplines, HMPs are physical practices embedded in holistic philosophies of well-being. Non-competitive- and non-performance-oriented in nature, HMPs’ integrative philosophies link movement forms and instructional frameworks not only to physical but also to mental, social, and/or spiritual well-being [5]. As such, HMPs go beyond traditional forms of exercise, frequently including an emphasis on somatic experiencing, body awareness, mindfulness, and/or personal growth. HMPs include traditional Eastern movement practices such as Yoga, Tai Chi, and Qigong, which have a long history with roots in India and China, but also a range of newer, “Westernborn” movement practices, for example, 5Rhythms, Biodanza, Nia, Pilates, and Eurythmy [5]. Research on health outcomes is extensive, and generally positive, for the traditional Eastern HMPs [6,7,8,9,10,11,12], and in its infancy, but still promising, for the newer Western-born HMPs [13,14,15,16,17,18].

When it comes to behavioural aspects, the traditional Eastern HMPs have received some attention in the form of participation prevalence, correlates, and motives. For example, in the USA, surveillance studies have shown steady increases in rates of Yoga participation, from 5.1% in 2002 to 13.3% in 2017, while prevalence for Tai Chi/Qigong remained stable at just over 1% [19,20]. Studies in both the USA and Australia indicate that levels of participation in Yoga are considerably higher than in Tai Chi/Qigong [19,21]. In Australia, recent surveillance data suggest Yoga is among the top 10 most practiced forms of physical activity in the country (https://www.clearinghouseforsport.gov.au/research/ausplay/results (accessed on 18 August 2021)). It has typically been found that the majority of Yoga participants are female, white, well-educated, and of higher socio-economic status [20,22], while participants in Tai Chi/Qigong show some overlap but also variation in characteristics, such as higher proportions of men, persons of Asian ethnicity, and non-university-educated [22,23]. Participation motives for Yoga and Tai Chi/Qigong are often initially health- and fitness-related, but may take on more holistic aspects such as calmness, self-knowledge, mind–body integration, and spirituality with longer engagement [24,25,26,27].

In contrast to those of traditional Eastern HMPs, participation parameters of Western-born HMPs have received very limited attention. Among early research is a qualitative study exploring the participation motives of 5Rhythms’ participants [28], which found that the holistic nature of the practice was reflected in motives such as emotional exploration and expression, safety, personal growth, and spirituality. A recent survey among 5Rhythms, Biodanza, and other free-movement-based holistic dance practices had a majority of female, white, and university-educated respondents, with many reporting a mental health condition [29]. In an Italian study, people signing up for a yearlong course in Biodanza were found to have different emotional characteristics than those signing up for other dance courses [16]. A survey among Brazilian Pilates participants showed the majority of respondents to be middle-aged women who did not participate regularly in other exercise activities and suffered some musculoskeletal pain [30]. While Pilates is sometimes included in physical activity surveillance studies [21,31], gauging participation information of smaller HMPs from surveillance studies is difficult, as they may be too small to capture and may end up being categorised as “other” or coded within wider categories such as dance or martial arts.

Surveillance and other surveys predominantly focus on individual-level factors influencing participation. However, ecological models suggest that there are multiple levels of influence on physical activity behaviour and that environmental contexts play an important role in shaping or constraining intra- and interpersonal determinants [32]. In particular, *behaviour settings*—the social and physical situations in which behaviour takes place—can promote certain behaviours while discouraging or prohibiting others. Various studies have shown positive relationships between physical activity participation and facility and/or resource provision [33,34,35,36], suggesting that degree of use is related to the degree of local availability, which, in turn, may be associated with neighbourhood characteristics [37]. Models of service utilisation also point to the enabling role contextual factors play in the use of services, emphasising such aspects as the amount, varieties, locations, structures, and distributions of provision [38]. It is thus not unreasonable to expect that the degree and nature of HMP provision may affect the uptake of these practices. One approach to begin to gauge the role and place of HMPs in the contemporary landscape of physical activities, therefore, is to look at the supply side of these practices. It is evident that without guided opportunities to get to know and engage in HMPs, participation is not possible. Hence, provision is an essential condition for participation. However, we currently know very little about the provision of HMPs.

The purpose of this study, therefore, was to explore the provision of HMPs. We did this by focusing on the area of Greater Melbourne. Melbourne is one of Australia’s largest and most physically active cities [39], with a rich cultural diversity. Because of these characteristics and through personal experience, we expected that Melbourne would house a variety of HMPs. We aimed to document numbers and event features (e.g., type, cost, duration, location, and target group) available online. A secondary purpose was to explore the neighbourhood characteristics associated with HMP offerings by linking the location data to selected sociodemographic variables derived from the 2016 Australian Census.

## 2. Methods

We employed an internet search through the Google search engine to locate information about HMP offerings in Melbourne. A criterion for a movement practice to be considered an HMP was an embedding in a holistic philosophy of wellbeing, and the existence of a teacher training system, indicating that a practice has spread beyond its original founder. A list of HMPs was drawn up based on previous publications and the authors’ own experiences in this field [5,40,41] and used as a basis for the search. This list consisted of 23 HMPs. For 6 of these 23, we found no websites indicating a presence in Greater Melbourne at the time of the search (Eutony, JourneyDance, Movement Medicine, PanEurythmy, Sign Chi Do, and Soul Motion). During our search, we became aware of five more practices that we considered HMPs that we had not originally included, and we searched separately for these. Altogether, we conducted searches for 28 HMPs, finding a presence in Greater Melbourne for 22 of them. It became clear early on that trying to document all events for Yoga and Pilates was not feasible. We therefore restricted the search for these practices to the area in which we had started, the centre of Melbourne (searching with the term “CBD”—central business district). For all other HMPs, the searches were conducted across the whole of Greater Melbourne.

Due to resource limitations, the searches were conducted in the springs of 2016 and 2017, between October and December. Some events were searched in 2016, others in 2017. Because of the exploratory nature of the study, we coded every event that was announced on a website, regardless of its date (i.e., sometimes events were announced several months in advance of the actual event, particularly for non-regular events).

### 2.1. Search Strategy

The primary search was performed by typing three key words for each HMP in the search engine: “name of the HMP”, “Melbourne”, and “Australia”. For Yoga and Pilates another key word was added—“CBD”. After reviewing all websites that listed all three/four key words, the search was repeated without “Australia”. When the search engine was no longer showing a connection between the two main key words “name of the HMP” and “Melbourne”, a secondary search was conducted. This included publicly open Facebook pages and websites that provided links to some other web or Facebook pages that were not initially found in the Google search.

### 2.2. Data Extraction

We focused on “events offered” as our unit of analysis. Events referred to any type of offering aimed at a group of people (i.e., not individual sessions), for example, classes or workshops. For each event mentioned on a website, the following information was recorded, if present on the site: event type, event name, location/venue address, venue type, day of the week, start time, duration, cost, level, and target group. If the venue type was not clear from the website the venue address was entered into the Google Maps search engine to determine more specifically what type of venue existed at the indicated location.

### 2.3. Association with Neighbourhood Demographics

Venue addresses were used to determine the postcode area in which an event was offered. Number of events were then determined per postcode area. We extracted a range of postcode-based socio-demographic variables from the 2016 Australian Census (https://datapacks.censusdata.abs.gov.au/datapacks/ (accessed on 2 November 2017)) and explored their relationship with HMP-offering postcode areas via correlations and *t*-tests.

### 2.4. Analyses

Descriptive statistics (mainly numbers and percentages) were used to analyse and report the data concerning event characteristics. For event duration and cost, means, medians, and standard deviations were determined. The association between HMP-offering postcode areas and selected socio-demographic census variables was explored via *t*-tests (HMP present/not present in the postcode area) and correlations (number of HMP events in a postcode area). Because of the exploratory nature of the study, we focused our description on characteristics that were significant at *p* < 0.01 for both the *t*-tests and correlations.

## 3. Results

### 3.1. Number of Events

For the 28 HMPs included, a total of 1483 events were found, representing 22 HMPs. This included 1011 events for Yoga and Pilates in central Melbourne, and 472 events for other HMPs across Greater Melbourne (Table 1).

To create some structure, we decided to present the results in three categories. Because a relatively large number of events was found for Tai Chi and Qigong, and these practices are closely related [42], we elected to split the HMPs in Greater Melbourne, with Tai Chi/Qigong presented separately and the remaining 18 HMPs referred to as “smaller HMPs”. Findings for Yoga and Pilates in central Melbourne are presented alongside. Table 2 provides an overview of the event characteristics described below.

### 3.2. Event Types

Event type was indicated for 99.3% of event offerings, with the great majority being weekly classes. For Yoga/Pilates in central Melbourne, virtually all events were weekly classes (99.3%) (Table 2). For Tai Chi/Qigong it was similar; most events were weekly classes (95.0%). However, for the smaller HMPs, although weekly classes made up more than half the events (59.7%), there was a greater proportion of less regular offerings in the form of one-off classes (18.8%), workshops (7.6%), seasonal events (8.3%), and monthly classes (5.6%).

Four of the smaller HMPs offered weekly classes only, while six did not offer weekly classes at all (Table 1 and Supplementary Table S1). Authentic Movement offered irregular (66.7%) and monthly (16.7%) classes only, as well as workshops (16.7%); Body-Mind Centering offered irregular classes (33.3%) and workshops (66.7%) only; Dances of Universal Peace and Gyrotonics did not mention type of event; Eurythmy offered irregular classes only; and Trance Dance either did not mention type of event or offered workshops (100%) only.

Workshops were found for 10 HMPs: 5Rhythms (4.3% of offerings), Authentic Movement (16.7%), Biodanza (33.3%), Body-Mind Centering (66.7%), Feldenkrais (6.1%), Open Floor (14.3%), Qigong (9.2%), Trance Dance (50%), and Yoga (0.5%).

### 3.3. Timing and Duration

#### 3.3.1. Duration

The duration of the sessions was not clear for 182 out of the 1483 events (12.3%). Overall, the mean duration of weekly classes was 57.2 ± 18.1 min. Monthly and irregular classes tended to be longer (123.0 ± 47.9 and 118.7 ± 42.3 min, respectively). Predictably, workshops were longest in duration (395.0 ± 256.0), but also varied considerably, ranging from 3 h half-day workshops to 18 h weekend workshops.

Weekly classes differed in mean duration between the categories of HMPs, with Yoga/Pilates classes being significantly (ANOVA, *p* < 0.001) shorter (53.3 ± 13.0 min) than classes for both Tai Chi/Qigong (67.9 ± 20.9 min) and the smaller HMPs (73.0 ± 34.7 min). The difference between Tai Chi and the smaller HMPs was not significant. The shorter duration for Yoga/Pilates was mainly due to shorter Pilates sessions (48.5 ± 7.2 min) compared to Yoga (61.3 ± 16.5 min). More than three quarters (76.7%) of all weekly Pilates classes were shorter than an hour; for Yoga, this was 26.4%. In contrast, for 20.3% of the smaller HMPs and 25.5% of Tai Chi/Qigong events, weekly classes were longer than 90 min.

#### 3.3.2. Time of Day

The starting time was indicated for 98.2% of events. We categorised starting time into a time-of-day variable as follows: early morning (6:00–8.59); morning (9:00–11.29); lunch time (11:30–13.29); early afternoon (13:30–14:29); afternoon (14:30–16:29); late afternoon (16:30–17:29); early evening (17:30–18:59); and evening (19:00–20:29). For both Tai Chi/Qigong and the smaller HMPs across Greater Melbourne, the most common time for offering sessions was the morning (41.7% and 36.9%, respectively), followed by the evening (21.9% and 26.2%) (See Table 2). In contrast, the most common time for Yoga/Pilates in central Melbourne was lunch time (30.4%), followed by early morning (22.9%) and early evening (22.5%).

#### 3.3.3. Weekdays

Day-of-the-week information was provided for 99.1% of events. All workshops took place during weekends, either on one day (28.6% for each day) or on both days (42.9%). One seasonal event (in Qigong) lasted five days during the workweek. Overall, 60% of monthly classes were on a weekend day, as were almost half of irregular/one-off classes (48.5%). In contrast, for weekly classes weekday offerings were more common, with, overall, Monday through to Thursday being the most popular days. For the smaller HMPs, Tuesday through Friday were the most popular days (72.8% of classes were offered on these days), with Saturdays and Sundays accounting for 13.6%. For Tai Chi/Qigong, the majority of the provision was Monday to Thursday (74.8%), with Monday being the most popular day (20.7%). However, there was also a reasonable provision on the weekend (16.8%), particularly Saturdays (see Table 2). For Yoga/Pilates in central Melbourne, the majority of weekly classes (78.0%) were offered Monday through Thursday, with very few classes during the weekend (9.0%).

### 3.4. Costs

Costs were only mentioned for 45.6% of the events offered. For 33.6% of the smaller HMPs, 72.3% of Tai Chi/Qigong, and 51.6% of Yoga/Pilates offerings, costs were not mentioned or not clear. For comparisons, we used the highest cost charged for a session, disregarding possible discounts for concessions or multi-class passes. It is worth noting that a variety of such options (multi-class passes, concessions, and memberships) were available across the events and HMPs.

#### 3.4.1. Weekly Classes

Costs for weekly classes varied considerably among HMPs (Table 3). Excluding free sessions, the lowest session cost was AUD 10 for the smaller HMPs and Yoga/Pilates and AUD 2 for Tai Chi/Qigong. The highest session cost was AUD 26 for the smaller HMPs, AUD 28 for Tai Chi/Qigong, and AUD 60 for Yoga/Pilates. Mean costs per hour were lowest for Tai Chi/Qigong (AUD 11.59 ± 5.95), followed by the smaller HMPs (AUD 18.19 ± 6.37), and highest for Yoga/Pilates (AUD 35.24 ± 16.22). Pilates was by far the most expensive type of HMP, based on both price per session (AUD 43.33 ± 11.26) and price converted to hourly costs (AUD 50.12 ± 12.03). Mean hourly costs were more than twice as high as the next most expensive HMPs, Hannah Somatics (AUD 23.40 ± 3.68), Yoga (AUD 22.84 ± 4.78), and Chi Ball (AUD 22.50 ± 2.74). The least expensive HMPs were Dancing Freedom (AUD 8.00 ± 0), Tai Chi (AUD 9.85 ± 5.98), Ageless Grace (AUD 10 ± 0), and Tensegrity (AUD 10 ± 0).

#### 3.4.2. Workshop Costs

Average costs for workshops ranged between AUD 65 and AUD 95 for half-day workshops, AUD 0 and AUD 105 for full-day workshops, and AUD 330 and AUD 675 for weekend workshops. Comparison between workshop prices is not straightforward, as there could be great variations in what is included (e.g., meals, overnight accommodation for weekend workshops), and we did not record this.

#### 3.4.3. Free Offerings

Out of the 681 events for which prices were indicated, 11 (1.6%) were offered for free: three for the smaller HMPs (two Feldenkrais and one Kundalini dance), six for Tai Chi/Qigong (four Tai Chi, two Qigong), and two for Yoga/Pilates (two Yoga, zero Pilates). The free events entailed either taster or trial sessions associated with subsequent (charged-for) longer offerings (Feldenkrais, Kundalini Dance, and Qigong), sessions associated with senior citizen organisations (e.g., Tai Chi, as part of membership of “University of the 3rd Age” (U3A), or part of a seniors’ festival), or sessions organised in public spaces (e.g., Tai Chi or Yoga offered at Federation Square, or Tai Chi in the park). Surprisingly, a one-day workshop in Yoga and a five-day course in Feldenkrais were also offered for free.

### 3.5. Target Level and Groups

#### 3.5.1. Level

A target level such as beginner or advanced was specified for less than half (46.2%) of the events (Table 4). For a large proportion of the smaller HMPs (75.2%), no level was indicated, and a further 19.5% explicitly stated that all levels were welcome. For 5.4%, a differentiation was made between beginner and non-beginner. Among Tai Chi/Qigong offerings, about 40% were aimed at a specific level, either beginner (26.3%) or intermediate/advanced (13.6%). For Yoga/Pilates in central Melbourne, about 32% specified different levels, including beginner (11.0%), non-beginner (14.2%) or intermediate/advanced (6.4%).

#### 3.5.2. Target Groups

We included information as “target group” when events were specifically and/or exclusively offered to individuals with particular characteristics (e.g., children, older adults, men, women, or specific health conditions), as well as when events were promoted as being especially suitable for individuals with particular characteristics. We found such target group specifications for only a minority (13.1%) of events (Table 4).

For the smaller HMPs, nothing was said about the target population for the majority of events (80.5%), and when something was said, it was most commonly that the events were for everyone, regardless of age, ability and/or fitness level (9.4%). A small number of gender-specific events were offered (6.0%). Of the nine events that specified gender, four were within 5Rhythms (one exclusively for men, one exclusively for women, and two for mothers and children) and four were within Kundalini Dance (all for women). Target groups associated with health or well-being were not mentioned for any of the smaller HMPs.

For Tai Chi/Qigong, no mention of target population was also the most common occurrence (75.5%). Where mentioned, age was the most common indicator (13.3%), including offerings for older adults (32.6% of specified age targets), children (20.9% of specified age targets), and teens/adults (44.2% of specified age targets). Target groups associated with gender or preferences were not mentioned for any of the Tai Chi/Qigong events, and only a very small proportion (4%) mentioned health conditions (arthritis). Out of 323 Tai Chi/Qigong events, only 24 (7.4%) were specifically targeted at older adults. All age-related indicators were for Tai Chi events only; no age-related indicators were found for Qigong events. Of the small number of specifications for Qigong, most asserted that Qigong was open to all ages and/or fitness levels.

For the Yoga/Pilates offerings in central Melbourne, the target group was mentioned for only 8.5% of events, with most of these targeting people with physical health or stress issues (4.3%). All references to physical problems were associated with Pilates offerings only, where examples were given of the types of problems that Pilates would be useful for, such as back and neck pain, sports injury, rehabilitation from musculoskeletal surgery, etc. References to suitability for stressed or busy individuals were found for both Yoga and Pilates. Appeals to people who might have a preference for particular experiences, e.g., “those who want a slower guided practice” or “those ready to challenge their fitness level” were found for Yoga only. Details about target group subcategories can be found in Supplementary Table S2.

#### 3.5.3. Venue Types

Venue indications were found for 98.5% of events. A wide range of venues were found in which HMPs were offered. We categorised these into 23 types (see Supplementary Table S3 for descriptions of the venue types). They included studios or centres dedicated to a single HMP, in particular Yoga and Pilates, or to multiple HMPs, and sometimes to dance/movement or martial arts. Other venues were associated with more general physical activity options, such as fitness and/or health clubs, sports, leisure, and recreation centres. There was also an assortment of venues that were not primarily associated with physical activity. These particularly included neighbourhood houses/community centres, community halls, and church halls, but also education establishments, clubs or society venues, senior citizen centres, and even a library and a seasonal bush camp. Additionally, some venues were associated with physical or mental health provision.

As can be seen in Table 2, the smaller HMPs used the widest variety of venues (20), followed by Tai Chi/Qigong (18). Yoga/Pilates in central Melbourne made use of only eight different types of venues. Among the smaller HMPs, there was quite a large spread across the range of venues, with the most common being Yoga studios or centres (14.8%), church halls (12.8%), and neighbourhood houses/community centres (9.4%). It is worth noting that dance–movement studios (6.0%) and residential homes (5.4%) were only found among the smaller HMPs.

The most commonly used venues for Tai Chi/Qigong were community centres (20.1%), church halls (13.9%), and community halls (12.4%). Various other venues were only or mostly found for Tai Chi/Qigong and not for the smaller HMPs or Yoga/Pilates in central Melbourne. These included martial arts schools (9.3%), rooms in primary or secondary schools (7.7%), and outdoor spaces (7.7%). It is worth mentioning that all of the offerings within martial arts schools were Tai Chi classes; Qigong was not offered within martial arts schools, underscoring the martial arts connection of Tai Chi [43]. On the other hand, holistic health centres were among the more common Qigong-offering venues.

The most commonly used venues for Yoga/Pilates in the inner city were fitness/health studios (24.5%), musculoskeletal-health-oriented establishments such as physiotherapy or sports injury clinics (19.4%), dedicated Yoga studios (19.2%), dedicated Pilates studios (18.1%), and body–mind fitness studios (a studio space offering a variety of HMP and fitness training options, not dedicated to one type of HMP) (15.6%). Dedicated Pilates studios were sometimes used for Yoga offerings (9.2% of use of Pilates studios), but dedicated Yoga studios did not offer Pilates. Virtually all (99.0%) of the offerings in musculoskeletal-health-oriented establishments were Pilates offerings.

### 3.6. Location and Neighbourhood Characteristics

The number of offerings per postcode was calculated for the smaller HMPs and Tai Chi/Qigong (as Yoga/Pilates were only examined in central Melbourne, postcode data were not meaningful).

#### 3.6.1. Location

Overall, HMP offerings were found in 123 (44.7%) out of 275 postcode areas. Tai Chi/Qigong were found in 105 (38.2%) and the smaller HMPs in 52 (18.9%) postcode areas. (The individual numbers for Tai Chi/Qigong and the smaller HMPs do not add up to the total number because several postcode areas offered both types.). The spread across inner, metropolitan, and outer regions of Greater Melbourne was different for the smaller HMPs and Tai Chi/Qigong (Supplementary Table S4). Taking into account the uneven distribution of postcode areas across the different regions of the city, for the smaller HMPs the percentage of HMP-offering postcodes was significantly (*p* < 0.001) higher than expected in inner and metropolitan Melbourne, and considerably lower than expected in outer Melbourne. There were no significant differences for Tai Chi/Qigong, suggesting a relatively evenly proportioned distribution.

#### 3.6.2. Neighbourhood Characteristics

To explore the relationship of (degree of) HMP presence in a postcode and socio-demographic characteristics of the postcode areas, we conducted both *t*-tests (comparing postcodes with to postcodes without any HMP offerings) and Pearson product-moment correlations (using the number of offerings per postcode) in relation to the census variables (Table 5). We will highlight the variables that showed both significant differences on the *t*-tests and significant correlations, using a minimum α level of 0.01. Compared to postcode areas not offering HMPs, those offering HMPs differed significantly on various dimensions of size, gender, education, marital status, ethnicity, household characteristics, employment status, religion, volunteering, and walkability (Table 5).

Characteristics associated with postcode areas offering smaller HMPs included: higher percentages of women and lower percentages of men; higher percentages of women in the labour force; higher levels of education; smaller household size; higher percentages of lone-person and group households, and lower percentages of family households; higher neighbourhood walkability scores; higher percentages of volunteers; higher percentages of never married and lower percentages of married individuals; higher percentages of de facto and lower percentages of registered marriages; and higher percentages of secular, other spiritual, or no religious beliefs, and lower percentage of Christianity.

Characteristics associated with postcode areas offering Tai Chi/Qigong included: larger population size; higher percentages of women and lower percentages of men; higher percentages of university graduates; higher percentages of group households; higher percentages of individuals holding Chinese ethnicity; and lower percentages of volunteers. It is noteworthy that age and SES were not significantly associated with HMP-offering postcode areas.

## 4. Discussion

In this paper, we attempted to shed some light on the provision of HMPs—forms of physical activity embedded in holistic philosophies of well-being [5]. While the traditional Eastern HMPs such as Yoga and Tai Chi have become relatively well established within the landscape of physical activities in modern-day societies, and participation parameters of these practices have been researched as forms of physical activity [21,22] or complementary medicine [19,44], a range of newer Western-born HMPs also exist, about which very little is known. In this study, we focused on provision, as this is, although not sufficient, at least a necessary precondition to enable participation in HMPs. The study showed that a metropolitan city can house a substantial number of different HMPs, although practices vary widely in the number of events on offer.

### 4.1. Level of Provision

It was evident that both Yoga and Pilates far outnumbered the provision of Tai Chi/Qigong, as the number of events for Tai Chi/Qigong across Greater Melbourne was lower than the number of events for Yoga and Pilates in central Melbourne only. This discrepancy mirrors the considerably lower national prevalence rates of Tai Chi/Qigong participation compared to Yoga/Pilates [21], supporting the notion of a link between provision and uptake. Tai Chi/Qigong provision, in its turn, was more widespread than the provision of the smaller HMPs. Below, we will first summarise the characteristics of the HMP events that we found before focusing the discussion on selected issues.

### 4.2. Summary of Findings on Event Provision and Neighbourhood Characteristics

#### 4.2.1. Findings for Tai Chi/Qigong

The following was characteristic for Tai Chi/Qigong events: (a) the large majority of offerings were weekly classes, which lasted on average 68 min, at an average cost of about AUD 13 per session; (b) sessions were most commonly offered during the morning; (c) about three quarters of the events were offered Monday to Thursday, with a reasonable provision on Saturday as well; (d) a wide range of venues were used, with neighbourhood houses, community halls, and church halls being the most common; (e) offerings were spread relatively evenly across regions in Greater Melbourne; and (f) offerings were more common in neighbourhoods with large population sizes, higher proportions of women, university graduates, and people with Chinese ethnicity. Despite considerable research emphasis on Tai Chi’s health benefits for older people [45,46], only a very small proportion of Tai Chi/Qigong offerings was specifically targeted at older individuals.

Some differences between Tai Chi and Qigong were evident. For example, average costs for Qigong classes were higher than for Tai Chi classes, and Tai Chi was often taught within martial arts schools, whereas Qigong sessions were never taught in martial arts settings but were more associated with holistic health centres.

#### 4.2.2. Findings for the Smaller HMPs

The following was characteristic for the smaller HMPs: (a) while a majority of offerings were weekly classes, other types of events were also relatively common, such as one off/irregular classes, workshops, and seasonal events; (b) sessions were most commonly offered during mornings and evenings; (c) weekly classes lasted an average of 73 min, at an average cost of about AUD 18 per session, and with the majority offered on weekdays; (d) target level or group was not indicated for the majority of events, and where it was, suitability was mostly defined as everyone being welcome; (e) a wide range of venues were used, with Yoga studios, church halls, and neighbourhood houses the most common; (f) offerings were mostly concentrated in inner and metropolitan Melbourne, and less so in outer Melbourne; (g) offerings were more common in neighbourhoods characterised by higher proportions of women, higher levels of education, higher percentages of singles, group households, de facto marriages, and people holding secular, other, spiritual, or no religious beliefs.

#### 4.2.3. Findings for Yoga/Pilates

The following was characteristic for Yoga/Pilates events in central Melbourne: (a) virtually all events were weekly classes, mostly offered at lunch time, early morning, or early evening during weekdays, particularly Monday to Thursday; (b) weekly classes lasted an average of 53 min, at an average cost of about AUD 33 per session, with Pilates classes generally being shorter and more expensive than Yoga classes; (c) target level was indicated for about half of the events offered, with “open to all levels” being the most common; (d) target group was only indicated for a small number of offerings, with suitability for physical health or stress issues emphasised most; and (e) a limited range of venues was used, with fitness/health centres, dedicated Yoga or Pilates studios, and musculoskeletal-health-oriented establishments being the most common.

### 4.3. Costs of Yoga and Pilates

The Yoga/Pilates provision that we assessed was most likely coloured by its location within central Melbourne, with offerings catering for people working in the central business district. Timing parameters may have been particularly affected by this. To what extent the higher costs found for Yoga/Pilates were also due to this location is a moot point. For the few other HMPs that offered events in both central Melbourne and other regions (Tai Chi, Qigong, and Feldenkrais), there were no significant differences in prices between central Melbourne and other areas, suggesting that the higher costs for Yoga/Pilates may have been due more to HMP type than to location.

Even taking into account a possible location bias, Pilates clearly stood out among the HMPs for having the shortest sessions and being the most costly, both in terms of session price and in terms of price per hour. Some of this may be due to Pilates classes often being offered for smaller numbers, and small group and clinical Pilates classes were indeed the most expensive offerings. However, even then, the average session (AUD 32.18) and hourly (AUD 40.14) costs for the remaining “standard” Pilates classes were still higher than for any of the other HMPs (AUD 20.51/AUD 20.13, respectively). It is interesting in this respect that in Sport Australia reports of the period 2016–2018, “cannot afford it/cannot afford transport” was the most cited reason for dropping out of Pilates (23%), while this was the third highest reason given for dropping out of Yoga (at 16%) and dance (at 9%) [31,47,48]. It is conceivable that the higher costs for Pilates are affected by Pilates’ affiliation with musculoskeletal health centres/clinics and a more medically trained workforce.

For Yoga, session costs may be influenced by their link with commercial studios and/or health and fitness clubs, as the majority of Yoga sessions were offered in HMP-orientated studios and fitness/health centres. Prominent use of Yoga studios and fitness gyms was also reported in a study among UK Yoga participants [49]. These findings may reflect Yoga’s more established identity as a form of physical activity, as well as a possible supply–demand dynamic, given that higher socio-economic status is common among Yoga participants.

### 4.4. Newness of the Smaller HMPs

Apart from Pilates, most of the Western-born HMPs are relatively new and still emerging. Concomitant with this status, the number of events for the smaller HMPs was relatively limited. Furthermore, compared to Tai Chi/Qigong and Yoga/Pilates, the proportion of weekly classes was considerably less and the proportion of less frequent offerings much higher. This may reflect the possibility that these HMPs are offered by a very limited number of teachers. However, there might also be a philosophical decision to focus on less frequent, but perhaps longer and more intense, events.

The limited distinctions made between beginner and advanced levels may also reflect an early developmental stage, with perhaps the use of small groups in which skill differences can be more easily addressed with individual participant attention. On the other hand, quite a few of these practices are free-movement based and, not requiring the acquisition of motor skills, can more easily accommodate a blend of experience levels. Notwithstanding, even for these practices, sessions for more advanced practitioners might at times be useful. Future research may investigate the delivery dynamics of these smaller HMPs in more depth.

### 4.5. Implications for Uptake

Given HMPs’ proven and potential physical and mental health benefits [5,12,18,29,50,51], they have a potential role to play in health promotion. It is thus important to consider the provision characteristics in light of implicit constraints and affordances and the effects on who might find their way to these practices. For example, the finding that weekday mornings were among the most common times for Tai Chi/Qigong and the smaller HMPs offerings suggests an inclination to make sessions available particularly to people with flexible work schedules, or those retired or not working. To what extent this is a matter of demand, access to suitable space, or preferences dictated by provider circumstances remains to be investigated.

It is also noteworthy that no indication of target level was given for over half of the events. This was the case even for HMPs such as Yoga/Pilates and Tai Chi/Qigong, where learning new motor skills is an essential ingredient, and one might expect that distinctions between beginner and more advanced classes would be useful. What the barriers are for teachers to provide separate classes deserves attention in future research, as well as to what extent having separate beginner classes acts as a facilitator to uptake.

A further notable finding was the extensive absence of information concerning target groups. This applied to both offering sessions exclusively for specific subgroups and encouraging specific subgroups to attend more general sessions. Where included, explicit mention of everyone being welcome was relatively common among Tai Chi/Qigong and the smaller HMPs. While this implies an open-minded and welcoming approach to anyone regardless of age, gender, fitness level, etc., at the same time, there may be an implicit reliance on those who will naturally find their way to these practices through personal networks and/or an affinity with holistic philosophies. It is likely that without explicit efforts to attract specific subpopulations, participants will be disproportionally from populations that are naturally attracted to physical activity (e.g., those more active in general, well-educated, and more affluent [52]) and to alternative approaches to health or spirituality (e.g., women, the well-educated [53,54]). Offering sessions at seasonal events with multiple activities or as part of a community centre program, as identified in this study, may help put HMPs on the radar of a wider group of people. It makes intuitive sense that targeted sessions will speak more to target groups. Men-only Yoga classes might entice more men into doing Yoga, although the receptivity to this may vary [55]. However, specific advertising within the target group circles would be needed to make the target population aware of classes and potential benefits.

A general question of importance to HMPs is what strategies (if any) are employed by providers to try and reach potential participants, in particular individuals who might be at risk of inactivity and/or of physical or mental health problems, and those without an existing affinity with holistic philosophies. Do providers have the willingness to attract such subpopulations? Bennie et al. [56] found that fitness industry professionals, including some Yoga and Pilates instructors, showed a moderate interest in training high health-risk populations; however, they might lack the necessary competency to work with such populations. Future research could look at the ambitions, recruitment strategies, and constraints employed and experienced by HMP providers, many of whom will be individual teachers, particularly for the smaller HMPs.

### 4.6. Venues/Facilities

Supporting the trend of diversification in physical activity settings over the last several decades [57,58], this study found that HMPs are offered in a wide range of venues, many not typically or primarily associated with sport or exercise. Providers used rooms in a variety of institutions, frequently making use of natural affiliations with those institutions in terms of philosophical (e.g., Qigong being offered in holistic healing centres, Eurythmy being offered in a Steiner School) or other (e.g., free-movement-based HMPs being offered in dance studios, Tai Chi in martial arts schools, and Pilates in physiotherapy practices) dimensions. The use of church halls may be reflective of an affinity with spirituality, common among HMPs. Such natural affiliations may help promote HMPs to people with similar interests. It is also noteworthy that some commercial studios cater to several HMPs, creating what could be termed a holistic movement hub, while others concentrate on one type of HMP.

### 4.7. Neighbourhood Characteristics

Differences in neighbourhood socio-demographics suggest that the appearance, and possibly growth, of HMPs may be more likely in some neighbourhoods than others. Natural affinities also appear present in these associations, such as a stronger presence of Chinese residents in Tai Chi/Qigong-offering neighbourhoods, which is in line with the finding of Birdee et al. [59] that being Asian was associated with Tai Chi/Qigong practice in the United States. A stronger presence of de facto (vs. registered) marriages, and of absent or alternative (vs. Christian) religious beliefs, suggests that non-traditional values may be more common in neighbourhoods where the smaller HMPs are offered. This fits with the notion that the underlying philosophies of HMPs often include non-mainstream conceptions of health and/or spirituality [5]. Though there was no association with age, group and lone-person households were more and family households were less common in HMP-offering areas, particularly for the smaller HMPs. This may also indicate an association with less traditional lifestyles. Positive associations with percentages of women and university degrees echo participant characteristics of the more established traditional Eastern HMPs [20,22,23], tentatively suggesting that other HMPs may cater to similar population subgroups. It remains to be established whether the neighbourhood socio-demographics of HMP-offering areas are indeed reflective of participant socio-demographics, but it seems a reasonable possibility.

What determines where HMP providers offer events deserves further study. It is plausible that a mix of factors will play a role, including the home neighbourhood of HMP teachers, the availability of suitable venues, as well as considerations regarding access to target populations and commercial issues.

### 4.8. Strengths and Limitations

To the best of our knowledge, this was the first study to examine details of the provision of HMPs. A particular strength of the study is the generation of participation-relevant information on the smaller HMPs, as there is very little information about participation parameters for these practices due to their relative newness and small sizes. By linking venue location to census data, the study also provided insight into sociodemographic influences on where HMPs may be offered.

The study was exploratory, making use of web-based information in an attempt to generate some insight into the characteristics of HMP provision. As such, it had several limitations. We will have missed offerings that were not announced online and publicly. This may include privately organised sessions with specific subpopulations, e.g., Tai Chi sessions for nursing home residents, or teachers sharing information about upcoming events via closed-group Facebook pages, or through a mobile telephone network such as WhatsApp). We also only searched during the period October–December—spring and early summer in Melbourne—which may have affected the number and types of events we found. A further limitation was that not all information was necessarily presented on the websites (e.g., costs), and resource limitations prevented further follow-up. The fact that costs are often not mentioned on a website is interesting in itself. Why this is and what the effects are on people’s inclination to attend advertised events would be an interesting topic for future study. The study focused on one metropolitan city in Australia, and results may not generalize to other cities or countries. As Melbourne is among the more active cities in Australia [39], it is plausible that less active cities may also house less HMPs. This is for future research to examine. Furthermore, we only searched for the HMPs we knew about at the time. Although we believe we captured a majority of HMPs, we have come across a few others since then that we did not include in our search and that may or may not have been present in Melbourne at this time, for example, Azul and PraiseMoves. As these are relatively new and originate from outside of Australia, however, their presence in Melbourne would likely have been small or non-existent and probably would have reflected the characteristics for the smaller HMPs. Last, we only used one search engine, Google. While this is one of the most commonly used search engines [60], it is a limitation in that different search engines may detect different content.

## 5. Conclusions

HMPs, and the variety of settings in which they are offered, exemplify the increasing diversity of physical activity opportunities in modern-day societies. This study provided some initial insights into participation potential of these practices by focusing on provision in a large Australian city. Particularly, it showed both overlapping and distinctive provision features among HMPs, associations with selected neighbourhood characteristics, and affinities between the holistic nature of HMPs, venue choice, and neighbourhood socio-demographics. Given HMPs’ potential for health benefits through both their physical nature and added holistic dimensions, the constraints and affordances impacting the spread of these practices, particularly the new and emerging Western-born HMPs, deserve further research attention.

## Figures and Tables

**Table 1:** Number of events found per HMP, and proportion of weekly classes versus other types of events.

	*n*	%	% Weekly Class/Other
***Yoga and Pilates in central Melbourne***	***1011***	***100.0%***	
Pilates	646	63.9%	100/0
Yoga	365	36.1%	98.1/1.9
***Other HMPs across Greater Melbourne***	***472***	***100.0%***	
5Rhythms	23	4.9%	39.1/60.9
Ageless Grace	5	1.1%	100/0
Authentic Movement	6	1.3%	0/100
Biodanza	6	1.3%	16.7/83.3
Body-Mind Centering	3	0.6%	0/100
Chakradance	12	2.5%	33.3/66.7
Chi Ball	6	1.3%	100/0
Dances of Universal Peace	1	0.2%	?/? *
Dancing Freedom	1	0.2%	100/0
Eurythmy	2	0.4%	0/100
Feldenkrais	33	7.0%	78.8/21.2
Gyrotonics	2	0.4%	?/?
Hanna Somatics	10	2.1%	40/60
Kundalini Dance	5	1.1%	60/40
NIA	24	5.1%	95.8/4.2
Open Floor	7	1.5%	50/50
Qigong	87	18.4%	83.7/16.3
Tai Chi	236	50.0%	99.1/0.9
Tensegrity	1	0.2%	100/0
Trance Dance	2	0.4%	0/100

* Question marks indicate that the proportion of weekly classes versus other types of events could not be calculated due to missing information concerning event type.

**Table 2:** Event characteristics for the smaller HMPs, Tai Chi/Qigong, and Yoga/Pilates.

	Greater Melbourne				Central Melbourne Only		Total	
	Smaller HMPs (*n* = 149)		Tai Chi/Qigong (*n* = 323)		Yoga/Pilates (*n* = 1011)			
	*n*	% *	*n*	%	*n*	%	*n*	%
***Event Types***								
Weekly class	86	59.7%	305	95.0%	1001	99.3%	1392	95%
Monthly (1 or 2×) class	8	5.6%	1	0.3%	1	0.1%	10	1%
One-off/irregular class	27	18.8%	2	0.6%	4	0.4%	33	2%
Workshop	11	7.6%	8	2.5%	2	0.2%	21	1%
Seasonal event	12	8.3%	5	1.6%		0.0%	17	1%
**Total**	**144**	**100%**	**321**	**100%**	**1008**	**100%**	**1473**	**100%**
*Missing*	*5*/*149*	*3.4%*	*2*/*323*	*0.6%*	*3*/*1011*	*0.3%*	*10*/*1483*	*0.7%*
***Time of Day***								
Early morning (6:00–8:59)	2	1.5%	15	4.7%	231	22.9%	248	17%
Morning (9:00–11:29)	48	36.9%	133	41.7%	90	8.9%	271	19%
Lunchtime (11:30–13:29)	8	6.2%	23	7.2%	306	30.4%	337	23%
Early afternoon (13:30–14:29)	13	10.0%	25	7.8%	18	1.8%	56	4%
Afternoon (14:30–16:29)	8	6.2%	8	2.5%	20	2.0%	36	3%
Late afternoon (16:30–17:29)	4	3.1%	3	0.9%	68	6.7%	75	5%
Early evening (17:30–18:59)	13	10.0%	42	13.2%	227	22.5%	282	19%
Evening (19:00–20:30)	34	26.2%	70	21.9%	48	4.8%	152	10%
**Total**	**130**	**100%**	**319**	**100%**	**1008**	**100%**	**1457**	**100%**
*Missing*	*19*/*149*	*12.8%*	*4*/*323*	*1.2%*	*3*/*1011*	*0.3%*	*26*/*1483*	*1.8%*
***Weekday (Weekly Classes)***								
Monday	11	13.6%	63	20.7%	197	19.7%	271	20%
Tuesday	15	18.5%	55	18.0%	190	19.0%	260	19%
Wednesday	15	18.5%	60	19.7%	196	19.6%	271	20%
Thursday	14	17.3%	50	16.4%	197	19.7%	261	19%
Friday	15	18.5%	26	8.5%	129	12.9%	170	12%
Saturday	8	9.9%	42	13.8%	59	5.9%	109	8%
Sunday	3	3.7%	9	3.0%	31	3.1%	43	3%
**Total**	**81**	**100%**	**305**	**100%**	**999**	**100%**	**1385**	**100%**
*Missing*	*5*/*86*	*5.8%*	*0*/*305*	*0.0%*	*2*/*1001*	*0.2%*	*7*/*1392*	*1%*
***Duration of Weekly Classes***								
Thirty minutes or less	2	2.7%	6	2.6%	20	2.1%	28	2%
Between 31–59 min	9	12.2%	10	4.3%	524	55.9%	543	44%
Between 60–89 min	48	64.9%	156	67.5%	361	38.5%	565	45%
Between 90–119 min	5	6.8%	54	23.4%	31	3.3%	90	7%
Two hours or more	10	13.5%	5	2.2%	2	0.2%	17	1%
**Total**	**74**	**100%**	**231**	**100%**	**938**	**100%**	**1243**	**100%**
*Missing*	*12*/*86*	*14.0%*	*74*/*305*	*24.3%*	*63*/*1001*	*6.3%*	*149*/*1392*	*11%*
***Target Levels***								
All levels ^1^	29	78.4%	25	16.2%	173	35.0%	227	33.1%
Beginner	3	8.1%	85	55.2%	111	22.5%	199	29%
Non-beginner ^2^	5	13.5%		0.0%	145	29.4%	150	22%
Intermediate/advanced		0.0%	44	28.6%	65	13.2%	109	16%
**Total**	**37**	**100%**	**154**	**100%**	**494**	**100%**	**685**	**100%**
*Missing*	*112*/*149*	*75.2%*	*169*/*323*	*52.3%*	*517*/*1011*	*51.1%*	*798*/*1483*	*54%*
***Target Groups***								
All	14	48.3%	23	29.1%	16	18.6%	53	27%
Age	3	10.3%	43	54.4%	1	1.2%	47	24%
Gender	9	31.0%		0.0%	4	4.7%	13	7%
Health/well-being		0.0%	13	16.5%	44	51.2%	57	29%
Preference	1	3.4%		0.0%	17	19.8%	18	9%
Other	2	6.9%		0.0%	4	4.7%	6	3%
**Total**	**29**	**100%**	**79**	**100%**	**86**	**100%**	**194**	**100%**
*Missing*	*120*/*149*	*80.5%*	*244*/*323*	*75.5%*	*925*/*1011*	*91.5%*	*1289*/*1483*	*87%*
***Venue Type***								
Arts/culture centre/house	9	6.2%	9	3.0%		0.0%	18	1%
Body–mind fitness studio	1	0.7%	3	1.0%	159	15.6%	163	11%
Bush camp	8	5.5%	4	1.3%		0.0%	12	1%
Church hall	19	13.1%	45	14.8%		0.0%	64	4%
Club/society	7	4.8%	18	5.9%		0.0%	25	2%
Community hall	6	4.1%	40	13.1%		0.0%	46	3%
Dance–movement studio	9	6.2%		0.0%		0.0%	9	1%
Education	9	6.2%	4	1.3%		0.0%	13	1%
Fitness/health centre	4	2.8%	1	0.3%	250	24.5%	255	17%
Holistic health clinic/centre	7	4.8%	11	3.6%	21	2.1%	39	3%
Leisure/recreation/sports centre	2	1.4%	14	4.6%		0.0%	16	1%
Library	1	0.7%	3	1.0%		0.0%	4	0%
Martial arts school		0.0%	30	9.8%		0.0%	30	2%
Mental/psychological services centre	4	2.8%	5	1.6%	5	0.5%	14	1%
Miscellaneous	8	5.5%	3	1.0%		0.0%	11	1%
Musculoskeletal health	3	2.1%		0.0%	196	19.4%	199	14%
Neighbourhood house	14	9.7%	65	21.3%		0.0%	79	5%
Outdoors/public		0.0%	12	3.9%	1	0.1%	13	1%
Pilates studio	3	2.1%		0.0%	185	18.1%	188	13%
Primary/secondary school	1	0.7%	25	8.2%		0.0%	26	2%
Residential home	8	5.5%		0.0%		0.0%	8	1%
Senior citizens		0.0%	13	4.3%		0.0%	13	1%
Yoga studio/centre	22	15.2%		0.0%	194	19.2%	216	15%
**Total**	**145**	**100%**	**305**	**100%**	**1011**	**100%**	**1461**	**100%**
*Missing*	*4/149*	*2.7%*	*18*/*323*	*5.6%*	*0*/*1011*	*0.0%*	*22*/*1483*	*1.5%*

* Percentages are produced from the available information (i.e., the categories add up to 100%), with the percentages of missing information (not clear or not mentioned) provided separately, below the total percentages. ^1^ “All levels” was coded when the words “all levels” or “any level” were used, when “no prior experience necessary” was stated, or when “both regular and beginner practitioners” were welcome (and no further levels were indicated). ^2^ Level indications were classed as “non-beginner” when some prior experience was required or recommended to attend the event.

**Table 3:** Costs for weekly classes, per session and per hour (in Australian dollars).

	Costs Per Session				Costs Per Hour			
	Mean Cost Per Session (s.d.)	*n*	Range	Median	Mean Cost Per Hour (s.d.)	*n*	Range	Median
***Smaller HMPs***	***19.18 (5.08)***	***61***	***0–26***	***20***	***18.19 (6.37)***	***55***	***0–34***	***18***
5Rhythms	19.25 (3.01)	8	15–25	20	16 (11.11)	8	10–34	10
Ageless Grace	10 (0)	2	10–10	10	10 (0)	2	10–10	10
Chi Ball	22.5 (2.74)	6	20–25	23	22.5 (2.74)	6	20–25	23
Dancing Freedom	20 (0)	1	20–20	20	8 (0)	1	8–8	8
Feldenkrais	19.5 (7.65)	18	0–25	21	20.22 (6.42)	15	0–27	20
Hanna Somatics	25.67 (0.58)	3	25–26	26	23.4 (3.68)	2	21–26	23
NIA	17.9 (1.41)	20	15–20	18	18.14 (1.49)	18	15–22	18
Open Floor	20 (0)	2	20–20	20	11.43 (0)	2	11–11	11
Tensegrity	15 (0)	1	15–15	15	10 (0)	1	10–10	10
***Tai Chi/Qigong***	***12.92 (6.48)***	***76***	***0–28***	***13***	***11.59 (5.95)***	***62***	***0–20***	***12***
Qigong	16 (6.15)	30	0–28	18	13.84 (5.19)	27	0–20	13
Tai Chi	10.91 (5.94)	46	0–20	12	9.85 (5.98)	35	0–20	11
***Yoga/Pilates***	***33.42 (13.01)***	***484***	***0–60***	***25***	35.24 * (16.22)	***429***	***0–67***	***27***
Pilates	43.33 (11.26)	245	24–60	50	50.12 (12.03)	195	25–67	50
Yoga	23.26 (2.99)	239	0–25	25	22.84 (4.78)	234	0–33	23
**Total**	**29.51 (13.99)**	**621**	**0–60**	**25**	**30.84 (16.97)**	**546**	**0–67**	**25**

* The mean hourly costs for Yoga/Pilates were higher than the mean session costs because many of the Pilates sessions were less than an hour long.

**Table 4:** Target level and group for the smaller HMPs, Tai Chi/Qigong, and Yoga/Pilates.

	Greater Melbourne				Central Melbourne Only		Total	
	Smaller HMPs		Tai Chi/Qigong		Yoga/Pilates			
	*n*	%	*n*	%	*n*	%	*n*	%
***Target Levels***								
Not mentioned	112	75.2%	169	52.3%	517	51.1%	798	53.8%
All levels ^1^	29	19.5%	25	7.7%	173	17.1%	227	15.3%
Beginner	3	2.0%	85	26.3%	111	11.0%	199	13.4%
Non-beginner ^2^	5	3.4%		0.0%	145	14.3%	150	10.1%
Intermediate/advanced		0.0%	44	13.6%	65	6.4%	109	7.3%
**Total**	**149**	**100.0%**	**323**	**100.0%**	**1011**	**100.0%**	**1483**	**100.0%**
***Target Groups***								
Not mentioned	120	80.5%	244	75.5%	925	91.5%	1289	86.9%
All	14	9.4%	23	7.1%	16	1.6%	53	3.6%
Age	3	2.0%	43	13.3%	1	0.1%	47	3.2%
Gender	9	6.0%		0.0%	4	0.4%	13	0.9%
Health/well-being		0.0%	13	4.0%	44	4.4%	57	3.8%
Preference	1	0.7%		0.0%	17	1.7%	18	1.2%
Other	2	1.3%		0.0%	4	0.4%	6	0.4%
**Total**	**149**	**100.0%**	**323**	**100.0%**	**1011**	**100.0%**	**1483**	**100.0%**

^1^ “All levels” was coded when the words “all levels” or “any level” were used, when “no prior experience necessary” was stated, or when “both regular and beginner practitioners” were welcome (and no further levels were indicated). ^2^ Level indications were classed as “non-beginner” when some prior experience was required or recommended to attend the event. N.B. Percentages presented in this table are produced from the total number of events, in order to highlight the low proportion of events where target levels and groups were indicated. In comparison, Table 2 includes percentages for target levels and groups from the provided information.

**Table 5:** Relationship of HMP-offering postcode areas with 2016 census variables.

	Smaller HMPs			Tai Chi/Qigong			Combined		
	*Mean*		Correlation	*Mean*		Correlation	*Mean*		Correlation
	Yes ^a^ (*n* = 52)	No (*n* = 222)	r ^b^	Yes (*n* = 105)	No (*n* = 169)	r	Yes (*n* = 123)	No (*n* = 151)	r
***Population Size***									
Total population (*n*)	18,321.87	15,663.94	0.009	21,533.46 ***	12,835.02	0.232***	20,511.59 ***	12,630.5	0.193 **
***Gender***									
Females (%)	51.43 **	50.34	0.123 *	51.06 ***	50.23	0.130 *	51.12 ***	50.09	0.166 **
Males (%)	48.58 **	49.68	−0.117	48.95 ***	49.8	−0.125 *	48.90 **	49.94	−0.160 **
***Age***									
Age (median)	38.58	39.26	−0.102	38.22	39.7	−0.083	38.29	39.81	−0.118
***Education***									
Undergraduate degree (%)	20.41 ***	13.57	0.328 ***	16.32 **	13.97	0.143 *	17.07 ***	13.08	0.278 ***
Postgraduate degree (%)	7.63 ***	4.38	0.347 ***	5.55 *	4.66	0.117	6.00 ***	4.18	0.266 ***
Graduate diploma/certificate (%)	3.06 ***	2.06	0.301 ***	2.34	2.19	0.061	2.50 **	2.05	0.198 **
Certificate (%)	10.34 ***	14.18	−0.234 ***	12.73	13.9	−0.080	12.30 **	14.38	−0.181 **
Advanced diploma (%)	7.73	7.87	−0.093	7.99	7.75	0.113	7.91	7.79	0.046
***Marital Status***									
De facto marriage (%)	4.68 ***	3.48	0.364 ***	3.85	3.62	0.107	4.06 **	3.42	0.266 ***
Single (%)	35.35 **	28.74	0.325 ***	31.12	29.29	0.098	31.97 **	28.38	0.240 ***
Married (%)	37.03 **	40.66	−0.276 ***	39.40	40.32	−0.078	38.85 *	40.88	−0.200 **
Registered marriage (%)	15.98 **	17.66	−0.259 ***	17.24	17.41	−0.053	16.95	17.66	−0.171 **
Separated (%)	2.08 *	2.40	−0.100	2.28	2.38	−0.045	2.25	2.41	−0.085
Widowed (%)	3.79	3.98	−0.069	3.88	3.99	−0.014	3.84	4.03	−0.046
Divorced (%)	6.33	6.57	−0.025	6.4	6.6	0.001	6.45	6.58	−0.012
***Ethnicity***									
Chinese (%)	8.07	5.91	0.070	8.28 **	5.1	0.122 *	7.88 **	5.04	0.134 *
Australian (%)	26.32	28.05	−0.069	26.53	28.46	−0.062	26.62	28.62	−0.084
Indian (%)	2.31 *	3.09	−0.100	3.4	2.66	0.087	3.17	2.76	0.022
***Household***									
Group households (%)	6.98 **	3.82	0.347 ***	5.26 *	3.89	0.142 *	5.56 ***	3.48	0.287 ***
Lone-person households (%)	27.13 ***	22.28	0.229 ***	24.05	22.68	0.109	24.73 **	21.95	0.201 **
Household size (n)	2.46 **	2.65	−0.201 **	2.59	2.62	−0.078	2.57	2.65	−0.162 **
Family households (%)	65.89 ***	73.90	−0.305 ***	70.69	73.43	−0.136 *	69.70 **	74.57	−0.261 ***
***Income***									
Weekly household income (median)	1742.5 **	1573.68	0.099	1618.38	1597.85	−0.009	1646.85	1572.21	0.041
***SES* ^c^**									
2011 IRSAD score	1055.48	1103.6	−0.022	1035.43	1131.15	−0.035	1038.37	1140.17	−0.040
***Employment***									
Women in labour force (%)	59.40 *	56.23	0.167 **	57.44	56.45	.065	57.99 *	55.89	0.135 *
Perc. employment to population females (%)	56.11 *	52.61	0.161 **	53.8	52.94	.053	54.42	52.33	0.122 *
Persons in labour force (%)	63.19	61.09	0.123 *	62.01	61.16	.054	62.37	60.77	0.105
Perc. employment to population persons (%)	59.63	57.29	0.118	58.14	57.48	.042	58.59	57.04	0.092
Men in labour force (%)	67.26	65.97	0.076	66.88	65.8	.054	67.03	65.55	0.081
Perc. employment to population males (%)	63.42	62.14	0.067	62.78	62.14	.035	63.02	61.86	0.062
***Religion***									
Secular/other Spiritual/No Religious beliefs (%)	40.38 ***	33.7	0.267 ***	35.82	34.44	0.089	36.80 **	33.49	0.204 **
Christianity (%)	41.82 **	46.45	−0.245 ***	45.06	45.9	−0.068	44.38	46.55	−0.176 **
Islam (%)	1.92 *	3.11	−0.061	2.31	3.24	−0.075	2.25	3.4	−0.091
Aboriginal, Sikh, or other (%)	0.67 **	1.07	−0.110	1.08	0.94	−0.020	0.99	0.99	−0.070
Buddhism (%)	2.75	2.85	−0.006	3.34 *	2.51	0.070	3.19	2.54	0.054
Hinduism (%)	1.56	1.97	−0.072	2.18	1.72	0.107	2.04	1.77	0.051
***Volunteering***									
Non-volunteers (%)	59.28	59.9	0.014	60.89 **	59.09	0.142 *	60.45 *	59.24	0.122 *
Volunteers (%)	18.67 ***	15.85	0.154 *	16.16	16.52	−0.010	16.72	16.11	0.068
***Walkability* ^d^**	*n* = 69	*n* = 297		*n* = 101	*n* = 245		*n* = 121	*n* = 225	
Walkability score	69.78 ***	53.20	0.308 ***	58.45	54.35	0.082	60.83 ***	52.70	0.213 ***

^a^ “Yes” indicates postcodes in which at least one HMP event was offered; “No” indicates postcodes where no event was offered; *t*-test significant differences with no HMP-offering postcodes are indicated by stars in this column. ^b^ r signifies the correlation with the number of events offered in a postcode area. ^c^ SES indicates socio-economic status. For this we used the Index of Relative Socio-economic Advantage and Disadvantage (IRSAD) from the 2011 Census. ^d^ Walkability scores for 2016/2017 were available per suburb rather than postcode area (https://www.walkscore.com/AU-VIC/Melbourne (accessed on 7 November 2017)), so these scores represent HMP offerings per neighbourhood rather than postcode area. *** Significant at *p* < 0.001, ** significant at *p* < 0.01, and * significant at *p* < 0.05.

## Data Availability

The data presented in this study are available on request from the corresponding author.

## Supplementary Materials

The following are available online at https://www.mdpi.com/article/10.3390/ijerph181910365/s1, Table S1: Event type percentages within HMPs, Table S2: Target groups with subcategories, Table S3: Description of venue types, Table S4: Percentage of HMP-offering postcodes in different regions of Greater Melbourne.
