# Supplementary material for: Diversification of Physical Activities: An Exploration of Provision Characteristics of Holistic Movement Practices in a Large Australian City
Source: Int J Environ Res Public Health. 2021 Oct 1;18(19):10365. doi: 10.3390/ijerph181910365 (PMC8507811; doi:10.3390/ijerph181910365)
Supplement: Supplementary file 1 [file ijerph-18-10365-s001.zip › Supplementary Table S1.pdf]

Supplementary Table S1

Event types per HMP

	weekly class		monthly (1 or 2x) class		one off/ irregular class		workshop		seasonal event		not mentioned		Total	
	n	%	n	%	n	%	n	%	n	%	n	%	n	%
5Rhythms	9	39.1%	2	8.7%	3	13.0%	1	4.3%	8	34.8%		0.0%	23	100%
Ageless Grace	5	100.0%		0.0%		0.0%		0.0%		0.0%		0.0%	5	100%
Authentic Movement		0.0%	1	16.7%	4	66.7%	1	16.7%		0.0%		0.0%	6	100%
Biodanza	1	16.7%	1	16.7%	2	33.3%	2	33.3%		0.0%		0.0%	6	100%
Body-Mind Centering		0.0%		0.0%	1	33.3%	2	66.7%		0.0%		0.0%	3	100%
Chakradance	4	33.3%	1	8.3%	6	50.0%	1	8.3%		0.0%		0.0%	12	100%
Chi Ball	6	100.0%		0.0%		0.0%		0.0%		0.0%		0.0%	6	100%
Dances of Universal Peace		0.0%		0.0%		0.0%		0.0%		0.0%	1	100.0%	1	100%
Dancing Freedom	1	100.0%		0.0%		0.0%		0.0%		0.0%		0.0%	1	100%
Eurythmy		0.0%		0.0%	2	100.0%		0.0%		0.0%		0.0%	2	100%
Feldenkrais	26	78.8%	1	3.0%		0.0%	2	6.1%	4	12.1%		0.0%	33	100%
Gyrotonics		0.0%		0.0%		0.0%		0.0%		0.0%	2	100.0%	2	100%
Hanna Somatics	4	40.0%		0.0%	6	60.0%		0.0%		0.0%		0.0%	10	100%
Kundalini Dance	3	60.0%		0.0%	2	40.0%		0.0%		0.0%		0.0%	5	100%
NIA	23	95.8%	1	4.2%		0.0%		0.0%		0.0%		0.0%	24	100%
Open Floor	3	42.9%	1	14.3%	1	14.3%	1	14.3%		0.0%	1	14.3%	7	100%
Pilates	644	99.7%		0.0%		0.0%		0.0%		0.0%	2	0.3%	646	100%
Qigong	72	82.8%		0.0%	1	1.1%	8	9.2%	5	5.7%	1	1.1%	87	100%
Tai Chi	233	98.7%	1	0.4%	1	0.4%		0.0%		0.0%	1	0.4%	236	100%
Tensegrity	1	100.0%		0.0%		0.0%		0.0%		0.0%		0.0%	1	100%
Trance Dance		0.0%		0.0%		0.0%	1	50.0%		0.0%	1	50.0%	2	100%
Yoga	357	97.8%	1	0.3%	4	1.1%	2	0.5%		0.0%	1	0.3%	365	100%
**Total**	**1392**	**93.9%**	**10**	**0.7%**	**33**	**2.2%**	**21**	**1.4%**	**17**	**1.1%**	**10**	**0.7%**	**1483**	**100%**
