# Supplementary material for: Diversification of Physical Activities: An Exploration of Provision Characteristics of Holistic Movement Practices in a Large Australian City
Source: Int J Environ Res Public Health. 2021 Oct 1;18(19):10365. doi: 10.3390/ijerph181910365 (PMC8507811; doi:10.3390/ijerph181910365)
Supplement: Supplementary file 1 [file ijerph-18-10365-s001.zip › Supplementary Table S2.pdf]

Supplementary Table S2

Target groups with subcategories

	smaller HMPs		TC/QG		YP only		Total	
	n	%	n	%	n	%	n	%
**NOT MENTIONED**	**120**	**80.5%**	**244**	**75.5%**	**925**	**91.5%**	**1289**	**86.9%**
**ALL**	**14**	**9.4%**	**23**	**7.1%**	**16**	**1.6%**	**53**	**3.6%**
All Ages	2	14.3%	5	21.7%	2	12.5%	9	17.0%
all ages & abilities	1	7.1%		0.0%		0.0%	1	1.9%
all ages & fitness	9	64.3%	18	78.3%		0.0%	27	50.9%
all fitness levels		0.0%		0.0%	14	87.5%	14	26.4%
everyone	2	14.3%		0.0%		0.0%	2	3.8%
**AGE**	**3**	**2.0%**	**43**	**13.3%**	**1**	**0.1%**	**47**	**3.2%**
adults		0.0%	1	2.3%		0.0%	1	2.1%
children	1	33.3%	9	20.9%		0.0%	10	21.3%
children & teenagers	1	33.3%		0.0%		0.0%	1	2.1%
older adults	1	33.3%	14[1]	32.6%	1	100.0%	16	34.0%
teens/adults		0.0%	19	44.2%		0.0%	19	40.4%
**GENDER**	**9**	**6.0%**		**0.0%**	**4**	**0.4%**	**13**	**0.9%**
men	1	11.1%				0.0%	1	7.7%
mothers & children	3	33.3%			1	25.0%	4	30.8%
pregnant women		0.0%			3	75.0%	3	23.1%
women	5	55.6%				0.0%	5	38.5%
**HEALTH/WELL-BEING**		**0.0%**	**13**	**4.0%**	**44**	**4.3%**	**57**	**3.8%**
older adults with health needs			10[1]	76.9%		0.0%	10	17.5%
physical problems			3	23.1%	18	40.9%	21	36.8%
stressed/busy				0.0%	26	59.1%	26	45.6%
**PREFERENCE**	**1**	**0.7%**		**0.0%**	**17**	**1.7%**	**18**	**1.2%**
various preferences	1	100.0%			17	100.0%	18	100.0%
**OTHER**	**2**	**1.3%**		**0.0%**	**4**	**0.4%**	**6**	**0.4%**
Chinese		0.0%			1	25.0%	1	16.7%
embodied practitioners	1	50.0%				0.0%	1	16.7%
sport		0.0%			3	75.0%	3	50.0%
other	1	50.0%				0.0%	1	16.7%
**Total**	**149**	**100.0%**	**323**	**100.0%**	**1011**	**100.0%**	**1483**	**100.0%**
