# Supplementary material for: Diversification of Physical Activities: An Exploration of Provision Characteristics of Holistic Movement Practices in a Large Australian City
Source: Int J Environ Res Public Health. 2021 Oct 1;18(19):10365. doi: 10.3390/ijerph181910365 (PMC8507811; doi:10.3390/ijerph181910365)
Supplement: Supplementary file 1 [file ijerph-18-10365-s001.zip › Supplementary Table S3.pdf]

**Supplementary Table S3**

Description of Venue Types

| | VENUE TYPE | Typically included: |
|---|---|---|
| 1 | Arts or Culture Centre/House | Community arts or culture centre; museum |
| 2 | Body-Mind Fitness Studio | Fitness studios that predominantly offer body-mind or mind-body practices (often Yoga/Pilates) but also include some other fitness activities, e.g. functional fitness |
| 3 | Bush Camp | Organised bush camps for families or groups |
| 4 | Church Hall | Halls or rooms in or associated with churches |
| 5 | Club/Society | Venues belonging to clubs or societies, e.g. youth clubs, scouts, angling club, sailing club, life saving club |
| 6 | Community Hall | Freestanding Halls or Pavilions available for community hire |
| 7 | Dance-Movement Studio | Dance-orientated studios or centres |
| 8 | Education | Non-primary/secondary school-education orientated institutions, e.g. Adult Education, Rudolf Steiner Training Centre, Environmental Education; Shiatsu College |
| 9 | Fitness/Health Centre | Fitness and Health-related centres or clubs, such as fitness centres, gyms, fitness studios, health clubs, city baths |
| 10 | Holistic Health Clinic/Centre | Clinics, Centres, or businesses focused on holistic health (e.g., acupuncture clinic; naturopathy; floors above holistic shops) |
| 11 | Leisure/Recreation/Sports Centre | Leisure, Recreation, or Sports centres |
| 12 | Library | Room or space in a library |
| 13 | Martial Arts School | School or Academy providing training in one or more of the martial arts |
| 14 | Mental/Psychological Services Centre | Centres focused on providing mental health or psychological treatment and/or services |
| 15 | Miscellaneous | A variety of venues difficult to classify, e.g. conference centre, medical clinic, Feldenkrais studio, Rehabilitation centre, Retreat House, Clothing store, Workshop & practitioner space |

| | | |
|---|---|---|
| 16 | Musculoskeletal Health | Clinics or centres focused on treatment of musculoskeletal problems, e.g., sports medicine, physiotherapy, osteopathy, massage, myotherapy |
| 17 | Neighbourhood House | Community centres, Community Houses, Neighbourhood Houses, or Neighbourhood centres; multicultural HUB, |
| 18 | Outdoors/Public Space | Outdoor spaces such as parks, gardens, or squares, or public indoor spaces such as Atrium |
| 19 | Pilates Studio | Studio spaces specifically designated as Pilates studios |
| 20 | Primary/Secondary School | Classrooms, gyms, or other spaces in either primary (most) or secondary (one) schools |
| 21 | Residential Home | Space used is at a residential address (e.g., home studio in garage) |
| 22 | Senior Citizens Centre | Senior citizens clubs, retirement villages, or veterans centres |
| 23 | Yoga Studio/Centre | Studios or Centres specifically designated for Yoga training |
