# Supplementary material for: Diversification of Physical Activities: An Exploration of Provision Characteristics of Holistic Movement Practices in a Large Australian City
Source: Int J Environ Res Public Health. 2021 Oct 1;18(19):10365. doi: 10.3390/ijerph181910365 (PMC8507811; doi:10.3390/ijerph181910365)
Supplement: Supplementary file 1 [file ijerph-18-10365-s001.zip › Supplementary Table S4.pdf]

Supplementary Table S4

Percentage of HMP-offering postcodes in different regions of Greater Melbourne

	Smaller HMPs			Tai Chi/Qigong			Combined[b]		
	**n**	**%**	**% dif[a]**	**n**	**%**	**% dif[a]**	**n**	**%**	**% dif[a]**
Inner MB	15	28.8%	17.6	17	16.2%	4.9	23	18.7%	7.4
Metropolitan MB	27	51.9%	14.8	41	39.0%	2	51	41.5%	4.4
Outer MB	10	19.2%	-32.4	47	44.8%	-6.9	49	39.8%	-11.8
Total	52	100%	0	105	100%	0	123	100%	0

[a] %dif = difference between found % and expected % ; if value is positive, the percentage of HMP-offering postcodes in that region is higher than expected based on a proportional representation; if negative, it is lower.

[b] combined is smaller than the sum of the smaller HMPs and Tai Chi/Qigong, because many postcode areas offered both practices.
